# Supplementary material for: Transcriptional Profiles Associated with Marek’s Disease Virus in Bursa and Spleen Lymphocytes Reveal Contrasting Immune Responses during Early Cytolytic Infection
Source: Viruses. 2020 Mar 23;12(3):354. doi: 10.3390/v12030354 (PMC7150966; doi:10.3390/v12030354)
Supplement: Supplementary file 1 [file viruses-12-00354-s001.pdf]

Supplemental material

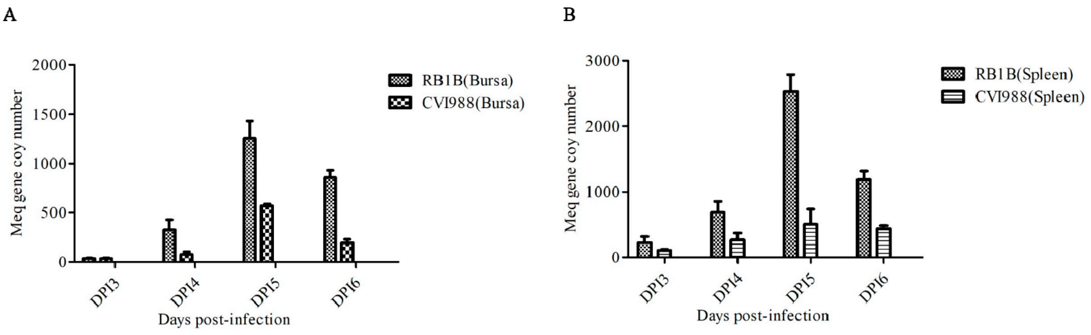

Figure S1 Replication of vvRB1B and CVI988 virus in splenic and bursal lymphocytes at different days of post-infection (dpi). A. The viral genome load in vvRB1B and CVI988 infected bursal lymphocytes. B. The viral genome load in vvRB1B and CVI988 infected splenic lymphocytes.

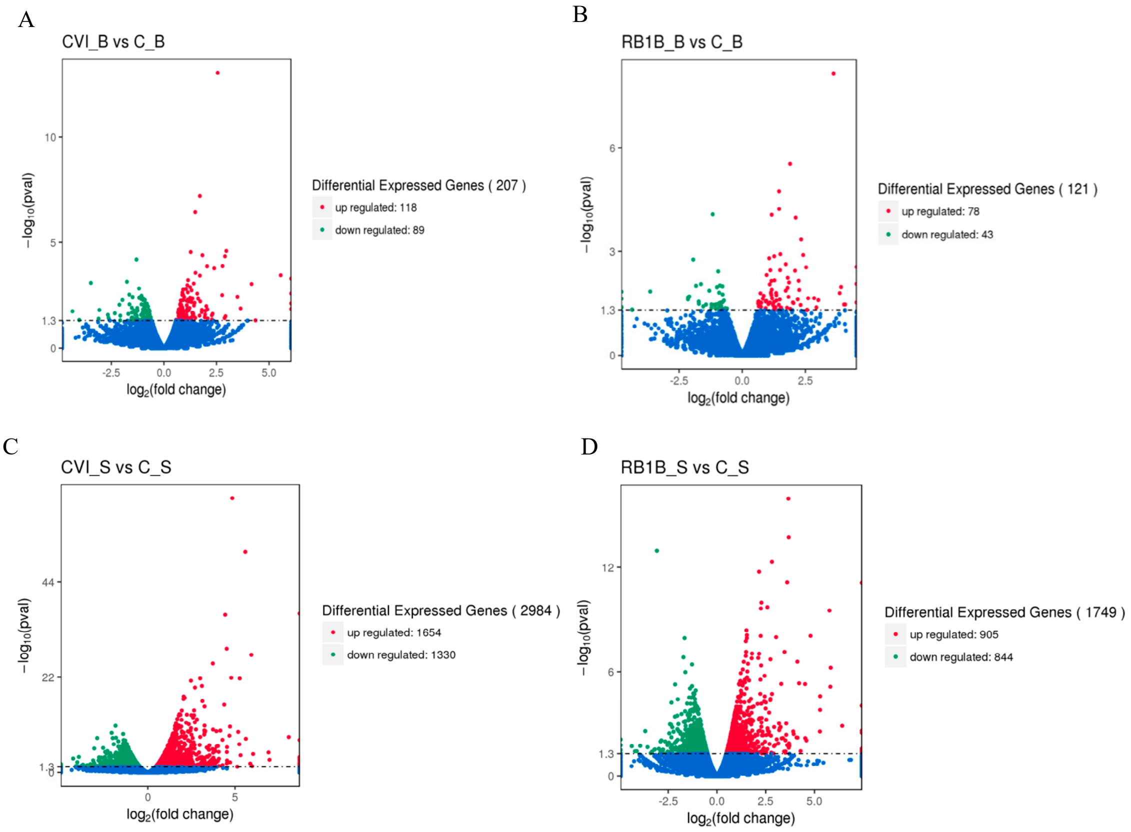

Figure S2 The overall distribution of differentially expressed genes of splenic and bursal lymphocytes demonstrated by the Volcano plot. Three individuals in each group were used for identifying DEGs. A. The expression of DEGs in bursal lymphocytes between CVI988 group and control group. B. The comparison of DGEs of bursal lymphocytes between the vvRB1B group and the control group. C. The expression of DEGs in splenic lymphocytes between CVI988 group and control group. B. The comparison of DGEs of splenic lymphocytes between the vvRB1B group and the control group. (The statistical criteria are p< 0.05 and ∣log2fold change > 1)

Table S1 Number of differentially expressed genes of splenic and bursa lymphocytes at dpi5.

Group<br>Criteria	C_B *VS* CVI_B(DPI5)	C_B VS RB1B_B(DPI5)	C_S *VS* CVI_S(DPI5)	C_S *VS* RB1B_S(DPI5)
p<0.05 ∣ FC∣>1	Up : 118<br>Down : 89	Up : 78<br>Down : 43	Up : 1654<br>Down : 1330	Up : 905<br>Down : 844
p<0.05 ∣ FC∣>1.5	Up : 36<br>Down : 22	Up : 36<br>Down : 11	Up : 408<br>Down : 226	Up : 189<br>Down : 124
p<0.05 ∣ FC∣>2	Up : 24<br>Down : 11	Up : 24<br>Down : 5	Up : 252<br>Down : 103	Up : 117<br>Down : 57

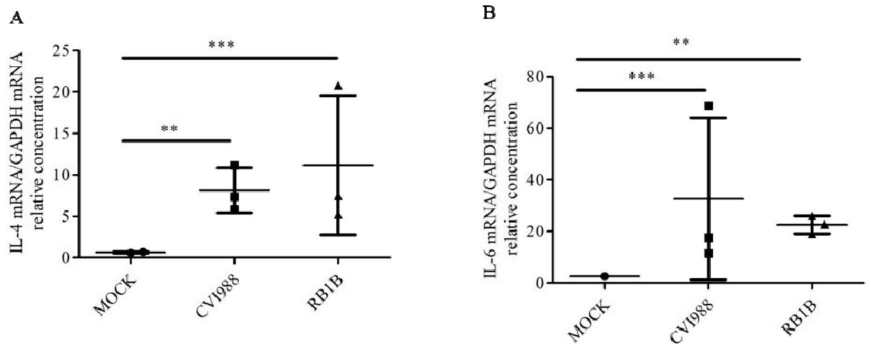

Figure S3 Profile of gene expression in bursal lymphocytes in CVI988, vvRB1B, and the control groups. The relative gene expression of IL-4 and IL-6 in CVI988, vvRB1B treated and control group bursal lymphocytes.
